# Abstract of the Proceedings of the Buffalo Medical Association

**Published:** 1863-12

**Authors:** J. F. Miner

**Affiliations:** Secretary


ART. II.—*Abstract of the Proceedings of the Buffalo Medical Association.*

TUESDAY EVENING, November 3, 1863.

Dr. Congar, President, in the Chair. The minutes of the last meeting read and approved.

*Dr. Rochester*, for the purpose of bringing the subject before the profession, would relate a case of delirium tremens. A young man, 26 years of age, called at his office, apparently rational. Said he had been drinking very badly, as many as twenty or thirty times in a day. Stomach was irritable, rejecting all injesta; tongue raw and red, with tenderness over the epigastrium; bowels loose, frequent mucous dejections. Thirst intolerable. Urine nearly natural. Directed sinapism to the epigastrium. Calomel ten grains, to be followed in four hours by Seidlitz powder, and in four hours another, if there was no dejection from the bowels; bland nourishment and as little stimulus as possible.

Next morning found he had retained the calomel and some food; bowels had moved. Thirst continued as urgent and stimulants strongly insisted upon by the patient. Gave milk freely with lime water, also soda water; whisky ℥i every two hours. Morphine gr, i every four hours, until he should obtain some sleep; blister to be applied over epigastrium.

Next morning had slept an hour, was as thirsty as ever, had rejected the soda water, but retained a little of the milk and lime water. The blister was now dressed with morphine; took nothing but ice, and refused everything else. Received notice this morning that he was dying; went to the hospital as soon as possible and found that he was dead. Treatment in the case was almost nugatory. Mentioned the case as one of fatal issue in first attack, He was confined to his bed, and some might think that his not being allowed to walk about the ward was injurious, but he would probably have died while walking. Dr. R. mentioned a case where a patient was walking about the ward; he lay down and in a few minutes afterwards died.

*Dr. Gay* mentioned a similar case of sudden death, which occurred in the General Hospital, Dr. Smith, house physician, giving him the particulars. The patient made a violent attempt to get out of the window; in this he was prevented, when he laid down, and was dead in a few minutes.

*Dr. Peters* would inquire if there was any remedy which would cure the appetite for liquor. Had seen nostrums advertised for its cure, but of course supposed there was nothing of it. Medicating spirits and giving great quantities had also been proposed for the cure of the appetite.

*Dr. Boardman* replied that the Physician in charge of the Asylum in Canandaigua said that they attempted the cure. That they watched carefully to exclude all stimulus and habituated them to life without it, encouraging their confidence in themselves. Mentioned cases which had been under his own care, and had suffered from repeated attacks of the disease, where, after the treatment, he had learned they even disliked the smell of spirits. Treatment consisted in purgations, Valerain, Peruvian Bark, &c.

*Dr. Peters* supposed that the same treatment pursued by the physicians in Canandaigua and by Dr. Boardman was essentially adopted everywhere. While physician to the Erie County Almshouse this general plan was adopted in great numbers of instances, but the same patients generally returned every year, and he thought they were *not cured.*

*Dr. Gay* thought amputation of the stomach was the only effectual remedy for its permanent cure.

*Dr. Rochester* remarked that similar cases to those reported by Dr, Boardman are known to all, but these are not usually cured, but the victims of delirium tremens almost always fill a drunkard's grave. He knew something of the Nostrum referred to by Dr. Peters; thought it unworthy a discussion in the Society. A great deal was claimed for it, as was usual with all Nostrums, but it was of no value whatever. In regard to curing by medicating spirits, or giving it in large quantities, he could only say, that generally they would take it, medicated or not, as much as they could get, and as long as they could get it.

*Dr. Boardman* said he was also of the opinion that those who have once delirium, almost always die drunkards.

*Dr. Gay* referred to the power of habit as illustrated also in those who chew tobacco, who can judge something of the strength of the influence. He believed that the taste once fully formed is in most instances so strong as to be impossible to reform.

*Voted,* on motion of Dr. Rochester that the "Special Subject" for next meeting be Delirium Tremens.

*Voted,* to adjourn.

J. F. MINER, Secretary.

